# Genetic relatedness to sisters' children has been underestimated

**DOI:** 10.1098/rspb.2012.1937

**Published:** 2013-01-22

**Authors:** Alan R. Rogers

**Affiliations:** Department of Anthropology, University of Utah, 270 S 1400 E, Salt Lake City, UT 84112, USA

**Keywords:** avunculate, genetic relatedness, paternity threshold

## Abstract

Males of many species help in the care and provisioning of offspring, and these investments often correlate with genetic relatedness. For example, many human males invest in the children of sisters, and this is especially so where men are less likely to share genes with children of wives. Although this makes qualitative sense, it has been difficult to support quantitatively. The prevailing model predicts investment in children of sisters only when paternity confidence falls below 0.268. This value is often seen as too low to be credible; so investment in sisters' children represents an unsolved problem. I show here that the prevailing model rests on a series of restrictive assumptions that underestimate relatedness to sisters' children. For this reason, it understates the fitness payoff to men who invest in these children. This effect can be substantial, especially in societies with low confidence in paternity. But this effect cannot be estimated solely from confidence in paternity. One must also estimate the probability that two siblings share the same father.

## 1. Introduction

In species with male parental care, it makes sense that males would direct that care towards offspring with whom they share genes. Male dunnocks, for example, often provision the young of several females, and the rate at which they provision each clutch closely matches their likely share of its paternity [1]. Similarly, many human males invest in the children of sisters rather than in those of wives, and this practice may be most widespread where extramarital mating is common [2–6] (but see [7–9]).

This human example was first approached in a quantitative way during the 1970s. In that decade, quantitative models were introduced by Alexander [10] and Greene [11,12]. I will refer to these as models A74, G78 and G80, respectively. Their common goal was to specify conditions under which men are genetically closer to sisters' children than to those of wives. The point at which they are equally close is called the ‘paternity threshold’ [11]. These authors assumed that selection would favour investment in sisters' children only when paternity confidence is below this threshold. The three models disagreed about its numerical value. The lowest value—0.268—was that of the G78 model. This model has become enshrined in the literature.

The others have been less influential. The A74 model was used in two publications [4,13] and the G80 model (without attribution) in one [14], but neither has been used since the 1980s.

The G78 model, on the other hand, continues to influence thinking. Anthropologists have extended it to various types of relative [2,15] and across several generations [5,6]. It also shows up in economic literature ([14]; [16], pp. 1923–1924). It is used in recent literature to argue that transfers to sisters' children compromise the reproductive interests of husbands ([17], p. 109; [18], p. 157).

It is not clear, however, that the paternity threshold provides a useful way to think about paternal investment. In the first place, it is hard to believe that paternity certainty is often as low as the model requires [4,5]. Furthermore, many forms of parental investment may exhibit decreasing returns to scale. In other words, the benefit from an additional unit of investment in any given offspring may decline with each unit invested. Where this is so, we might expect men to distribute investment among multiple offspring, weighting that investment in favour of those with whom they are most likely to share genes. Furthermore, the optimal behaviour of males will depend in part on the responses of females. Neither of these complexities is accomodated by the paternity threshold model.

They are central, however, to recent game theoretic analyses [19,20]. Those show that selection can favour investment in sisters' children even when paternity confidence is well above the paternity threshold. Yet, these models also use the G78 model in calculating fitness payoffs ([19], supplementary material, p. 8; [20]). That model thus continues in importance.

In what follows, I argue that all three models (A74, G78 and G80) make restrictive assumptions. Some of these have been acknowledged [4,11,12], but others seem not to have been noticed. All of them distort our views about the relatedness of men to sisters. These distortions may have biased the conclusions of all the work mentioned earlier.

This article will not try to explain investment by males in sisters' children. It will deal only with questions of relatedness, a more limited project that may prove useful in the larger one.

## 2. Material and methods

Most quantitative work on this subject has used the *coefficient of relatedness*. I work instead with the *coefficient of kinship* ([21], p. 121)—the probability that two genes, drawn at random from each of two relatives, are copies of the same gene in some given ancestral generation. In the absence of inbreeding, the coefficient of kinship equals half the coefficient of relatedness. We are interested in two of these coefficients, which describe the genetic kinship of a man to the children of (i) his wife and (ii) his sister.

These coefficients depend on two probabilities, which may vary among families. The first of these—*p*, or paternity confidence—is the probability that a child's father is his mother's husband. The other is *h*, the probability that two siblings share the same father. The value of *h* is relevant because it affects the genetic relatedness of a man (Ego) to his sister's children. This value depends on paternity confidence, but not that provided by Ego's wife. It depends instead on the paternity confidence provided by Ego's mother to her husband. The value of *h* also depends on the number of a woman's husbands [20]. I assume throughout that women have only one. Finally, I assume that Ego does not know values specific to his own family and must rely instead on population averages, 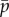 and 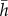.

**Figure 1.**
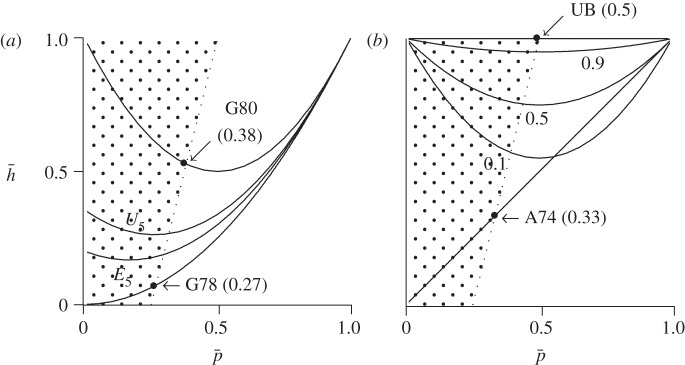
Effect of average paternity confidence 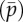 on the average probability 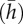 that two sibs share a father. In the shaded regions, men share more genes with the child of a sister than with that of a wife. Paternity thresholds of several models are indicated by filled circles. For these thresholds, the value of 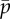 is shown in parentheses. (*a*) Paternity confidence constant across families. Curves G78 and G80 represent the models of Greene [11,12]. The other two curves assume that women have five extrapair mates, with whom the frequency of mating is either even (*E*_5_) or uneven (*U*_5_). (*b*) Paternity confidence varies among families. Curve A74 shows the Alexander model. The other curves assume that *b* = 1 and that, for each family, *p* is drawn from a Beta distribution with mean 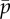. Labels show the variance of this distribution as a fraction of the maximum possible variance, 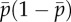. Curve UB shows the upper bound, at which the paternity threshold reaches its maximal value, 0.5.

Within a family, the coefficient of kinship of Ego with his wife's child is 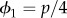, whereas that with his sister's child is 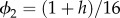. A man shares more genes with the child of a sister than with that of a wife when 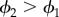, or equivalently when 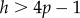. This condition refers to an individual family, but also holds on average if *p* and *h* are replaced by 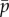 and 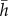. The shaded regions in figure 1 show the combinations of 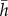 and 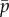 that satisfy this inequality.

Consider a family within which the probability of paternity is *p*. With probability *p*^2^, two random siblings were fathered by the husband, and with probability (1−*p*)^2^ neither was. In the second case, both may have been fathered by the same extrapair^1^ male. Let *b* represent the conditional probability of this event, given that neither sib was fathered by the husband. With these definitions,2.1
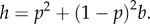


For an average family,
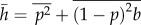
2.2
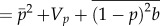
where the overbars represent averaging over families, and 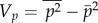 is the variance among families in *p*.

## 3. Results

This section will (*a*) derive the assumptions that underlie the three published models, and then relax assumptions involving (*b*) the distribution of sexual access among extrapair mates and (*c*) the variation of paternity confidence among families.

### (a) Assumptions of the classical models

The three models make differing claims about 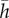:3.1
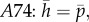
3.2
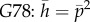
and3.3
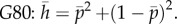


Each of these results can be derived by setting 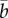 and *V_p_* to values at the edges of their feasible ranges—that is, by setting each parameter either to its highest or its lowest feasible value.

Because *b* is a probability, it must lie between 0 and 1, and so must its average, 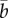. This average equals 0 when women never mate with the same extrapair male twice, or (equivalently) when each woman has an infinite number of such mates. On the other hand, 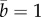 when no woman has more than a single extrapair mate. The variance, *V_p_*, must lie between 0 and 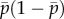. It attains the lower value when all women have the same fraction of extrapair matings. The upper value occurs when *p* = 1 for a fraction 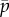 of women (who always mate with their husbands), and *p* = 0 for the rest (who never do). In summary, 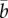 lies within the range [0,1] and *V_p_* within 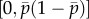.

Consider what happens when we substitute these upper and lower feasible values into equation (2.2). The result equals equation (3.1) when 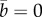 and 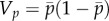, equals equation (3.2) when 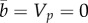, and equals equation (3.3) when 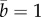 and *V_p_* = 0. Thus, models A74, G78 and G80 can each be derived by setting 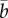 and *V_p_* equal to values at the limits of their feasible ranges. The A74 model also holds if 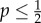 and *b* = *p*/(1 − *p*) within each family, for then equation (2.1) reduces to *h* = *p*, and averaging over families gives 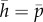 (equation (3.1)).

These results are summarized in table 1. For the G78 model, the assumptions discussed above are necessary as well as sufficient. Those for the A74 and G80 models are sufficient, but may not be necessary: they may hold also under other assumptions. None, however, have ever been described. The assumptions discussed earlier are the only ones under which these models are known to hold. Let us ask now what happens when these assumptions are relaxed. §3*b* considers the possibility that siblings share paternity through an extrapair male.

**Table 1.** Assumptions that underlie each model. The models differ with respect to assumptions about two parameters: *V_p_* (the variance across families in paternity confidence) and 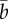 (the mean probability that two siblings share an extrapair father, if neither was fathered by the mother's husband). The first of these ranges from 0 to 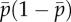 and the second from 0 to 1. Each model can be derived by setting each parameter either to its highest or its lowest feasible value. The A74 model can also be derived from assumptions that imply intermediate values of the two parameters. ‘Upper bound’ (UB) is the model with the highest possible paternity threshold.

		assumption	
model	abbreviation	*V_p_*	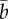
Greene [11]	G78	0	0
Greene [12]	G80	0	1
Alexander [10]	A74	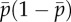	0
upper bound	UB	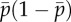	1

### (b) Extrapair paternity

This section focuses on the probability, *b*, that two siblings share a father, given that neither was fathered by their mother's husband. It will be useful to simplify the other parts of the model; so let us follow Greene [11,12] in assuming that *p* and *b* are constant across families. In this context, there is no distinction between values for families and averages across families; so I omit the overbars.

It is easy to derive the minimum and maximum feasible values of *h*. Equation (2.1) implies that *h* increases with *b*, for any given *p*. Consequently, the minimum *h* occurs when *b* = 0 and the maximum when *b* = 1. These correspond to curves G78 and G80 in figure 1*a*. These curves provide lower and upper bounds on the value of *h*, provided that *p* and *b* are constant across families. When families vary, however, we will see below that 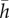 can be even larger than G80 would imply.

Between the two extremes, *b* = 0 and *b* = 1, the value of *b* will vary in response to the number of extrapair partners and the distribution of matings among them. To model this effect, let *g_i_* represent the fraction (among all extrapair matings of a given woman) of matings with the *i*th male. Then *g_i_*^2^ is the probability that two sibs were both fathered by this male, if neither was fathered by the husband. In these terms,3.4
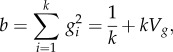
where *k* is the number of males, 1/*k* is the mean of the *g_i_* and 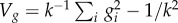 is the variance.

If the wife visits extrapair partners with equal frequency, then *b* = 1/*k*, and 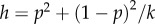. This reduces to model *G*80 if the wife has just a single extrapair partner, and to model G78 if she has an infinity of them. In addition to these curves, figure 1*a* also includes model *E*_5_, in which the wife allocates matings evenly among five extrapair partners. For a given value of *p*, the figure shows that *h* declines as sexual partners become more numerous, because offspring are then less likely to share paternity through an extrapair male.

When extrapair males receive uneven allocations, *V_g_* is large, increasing both *b* and *h*, and making siblings more similar. This makes intuitive sense: variation in *g_i_* implies that a small number of males enjoy disproportionate mating success; so random pairs of offspring are likely to share paternity through one of these favoured males. To illustrate this effect, figure 1*a* includes curve *U*_5_, representing the case of five extrapair males who get sexual access in proportion to 1, 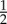, 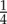, 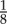 and 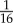. Because of this unevenness, curve *U*_5_ is higher than *E*_5_.

As each of the curves in figure 1*a* passes from the shaded to the unshaded region, 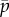 passes what Greene ([11], p. 153) called the ‘paternity threshold’—the ‘*p* below which a male is *more* related to his sister's offspring than to his spouse's’. Because of the slope of the boundary, the smallest and largest thresholds are those for the lowest and highest curves on the page. These two extremes—models *G*78 and *G*80—imply thresholds of 0.268 [11] and 0.382 [12].

So far, we have seen that relatedness to sisters (and thus to sisters' offspring) increases if the mother has a small number of extrapair partners or allocates matings unevenly among them. Furthermore, the figure shows that these effects can be quite large. §3*c* considers another influence—variation among families in paternity confidence.

### (c) Variation in paternity confidence

The fraction of children fathered by the current husband will ordinarily vary among families. In such cases, *p* and *h* are random variables, and attention turns to their averages, 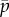 and 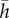. To obtain an upper bound on 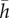, assume that *b* = 1 for all women. Then equation (2.2) becomes
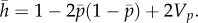


When the variance (*V_p_*) among families is large, 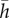 will also be large. To illustrate this effect graphically, I assume that *p* is drawn randomly for each family from a beta distribution with mean 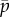. In figure 1*b*, the curves labelled 0.1, 0.5 and 0.9 refer to models with increasing variance among families. The larger the variance, the higher the probability 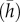 that two siblings share a father.

The maximal value of 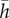 occurs when *b* = 1 for all women, and *V_p_* is at its maximal value, 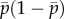. In this case, 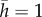, whatever the value of 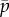. The paternity threshold attains its largest possible value, 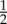. This model is shown as curve UB in figure 1*b*.

## 4. Discussion and conclusions

It has been understood for four decades that paternity certainty increases the relatedness of men to the children of wives and sisters [10]. But relatedness to sisters' children also responds to other influences, which have not been appreciated. It is greater when women have few extrapair partners and allocate matings unevenly among them, and when paternity confidence varies among families. Published models involve restrictive assumptions about all these influences.

These assumptions underlie an old debate about the correct form of the relationship between paternity certainty and relatedness to sisters' children. Various authors disagreed about which functional form was correct ([11], p. 153; [2], pp. 151–152; [13], p. 321; [4], pp. 443–444). The present work shows that all are correct—they simply involve different assumptions.

When spelled out, these assumptions seem remarkably restrictive. They include: (i) that paternity confidence is the same in each family (G78, G80); (ii) that women never mate twice with the same extrapair male (A74, G78); (iii) that some women always mate with their husbands, but the rest never do (A74); and (iv) that no woman mates with more than one extrapair male (G80). Each of these assumptions is unrealistic, and each biases estimates of genetic relatedness. The assumptions (i) and (ii) of the G78 model both bias results downward. Consequently, this model provides only a lower bound on the relatedness of men to sisters' children. Current theory relies on this model, and thus underestimates the fitness payoff to males who invest in such children. The upper bound on relatedness occurs when assumptions (iii) and (iv) both hold. At this upper bound, all siblings share paternity, because no sibship has more than one biological father.

Presumably, real populations lie somewhere between these extremes. This range of uncertainty implies that there is no single paternity threshold—no single value of 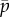 at which men are equally related to children of wives and of sisters. Instead, the paternity threshold varies among populations between 0.268 (the lower bound) and 0.5 (the upper). Even under the most generous conditions, the paternity threshold requires a very low confidence in paternity.

The present results also bear on recent game-theoretic analyses. Fortunato & Archetti [19] studied the evolution of monogamous marriage and ‘vertical transfer’ (i.e. investment in the children of wives). In calculating fitness payoffs, they used the assumptions of the G78 model. As we have seen, this minimizes the payoff from investing in sisters' children. Consequently, their model may overstate the stability of vertical transfers. Similarly, Fortunato [20] shows that selection can favour investment in sisters' children even when 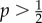. This conclusion is conservative because it also relies on the G78 model. Investment in sisters' children would evolve even more easily if the assumptions of this model were relaxed.

We can estimate men's kinship, 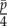, to wives' offspring directly from the population-wide average paternity confidence, 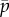. But we cannot estimate kinship to sisters' children from this value alone. It may lie anywhere between the lower and upper bounds, 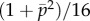 and 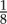. The difference between these bounds decreases with increasing values of 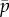. Among the Himba, for example, Scelza [22] estimates that 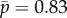. This implies that kinship to sisters' children is between 0.106 and 0.125, a range of 18 per cent. Had 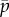 been lower—say 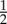
—the range of uncertainty would have been 60 per cent.

Without measuring, one cannot know where within this range of uncertainty any real population lies. To remove the uncertainty, we need separate estimates of paternity confidence 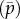 and of the probability 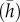 that two siblings share a father.

## Endnote

1 I use the term ‘extrapair’ rather than ‘extramarital’, because relationships that produce children need not be sanctioned as marriages. Nonetheless, I assume that one male is primary and refer to him as the ‘husband’.
